# Biochemical exploration of β-lactamase inhibitors

**DOI:** 10.3389/fgene.2022.1060736

**Published:** 2023-01-17

**Authors:** Varshaa Arer, Debasish Kar

**Affiliations:** Department of Biotechnology, Ramaiah University of Applied Sciences, Bangalore, India

**Keywords:** β-lactam, β-lactamases, mode of action, resistance, inhibition, inhibitors

## Abstract

The alarming rise of microbial resistance to antibiotics has severely limited the efficacy of current treatment options. The prevalence of β-lactamase enzymes is a significant contributor to the emergence of antibiotic resistance. There are four classes of β-lactamases: A, B, C, and D. Class B is the metallo-β-lactamase, while the rest are serine β-lactamases. The clinical use of β-lactamase inhibitors began as an attempt to combat β-lactamase-mediated resistance. Although β-lactamase inhibitors alone are ineffective against bacteria, research has shown that combining inhibitors with antibiotics is a safe and effective treatment that not only prevents β-lactamase formation but also broadens the range of activity. These inhibitors may cause either temporary or permanent inhibition. The development of new β-lactamase inhibitors will be a primary focus of future research. This study discusses recent advances in our knowledge of the biochemistry behind β-lactam breakdown, with special emphasis on the mechanism of inhibitors for β-lactam complexes with β-lactamase. The study also focuses on the pharmacokinetic and pharmacodynamic properties of all inhibitors and then applies them in clinical settings. Our analysis and discussion of the challenges that exist in designing inhibitors might help pharmaceutical researchers address root issues and develop more effective inhibitors.

## Introduction

Antibiotics are the single most consequential drug in the history of medicine. However, they are losing their potency as microorganisms evolve resistance (Babic et al., 2006). A substantial challenge to healthcare is the spread of multidrug-resistant (MDR) bacteria. A bleak picture of the establishment of β-lactam resistance is supported by numerous surveys that these mechanisms include changes in the permeability of the cell membrane and the intrinsic activity of efflux pumps; together, these limit drug absorption, alter drug targets and render antibiotics ineffective (Wanda 2018). In gram-negative bacteria, the emergence of β-lactamases is one process through which they acquire resistance to antibiotics (Watkins et al., 2013). In clinical practice, β-lactams—which impair the functionality of β-lactams—with antibiotics have proven to be the most successful approach, since they are efficacious with some controllable side effects (Mojica et al., 2022). Although these have long been studied, their evolution and behavior remain key scientific topics that require further inquiry (Stanton et al., 2020).

The bacteria of the Enterobacteriaceae family—*Pseudomonas aeruginosa*, *Neisseria gonorrhoeae*, *Mycobacterium tuberculosis*, and *Haemophilus influenzae*—are some of the prevalent organisms medicated by β-lactamase inhibitors (Curello and MacDougall, 2014). Certain Enterobacteriaceae like *P. aeruginosa* have acquired extended-spectrum β-lactamase enzymes (ESBLs), which provide additional resistance to cephalosporin antibiotics. According to research, β-lactamase inhibitors can effectively inhibit the synthesis of ESBL, therefore boosting its ability to destroy these dangerous pathogens (Tamma and Villegas, 2017). To maximize their efficiency, β-lactams must be used in conjunction with β-lactamase inhibitors. Many studies have attempted to find new and effective β-lactamase inhibitors (Khanna and Gerriets, 2022).

## Generic mechanism

An effective approach to inhibiting β-lactamase-mediated resistance is to design a drug that targets the active site of the enzyme. Inhibition can occur as either reversible or irreversible substrate binding with the formation of an acyl intermediate that results in steric interactions or activation of irreversible mechanisms (Bush, 1988). Reversible inhibition is analogous to the enzyme-substrate reaction. Through further chemical interactions at the enzyme active site, irreversible “suicide inhibitors” can irrevocably neutralize β-lactamase, as shown in Eq. 1.

$$E+I\begin{array}{l} k1\\ \Leftrightarrow \\ k_{-1} \end{array}E:I\overset{k2}{\to }E-I\overset{k3}{\to }EI*$$ (1)

**FIGURE 1:**
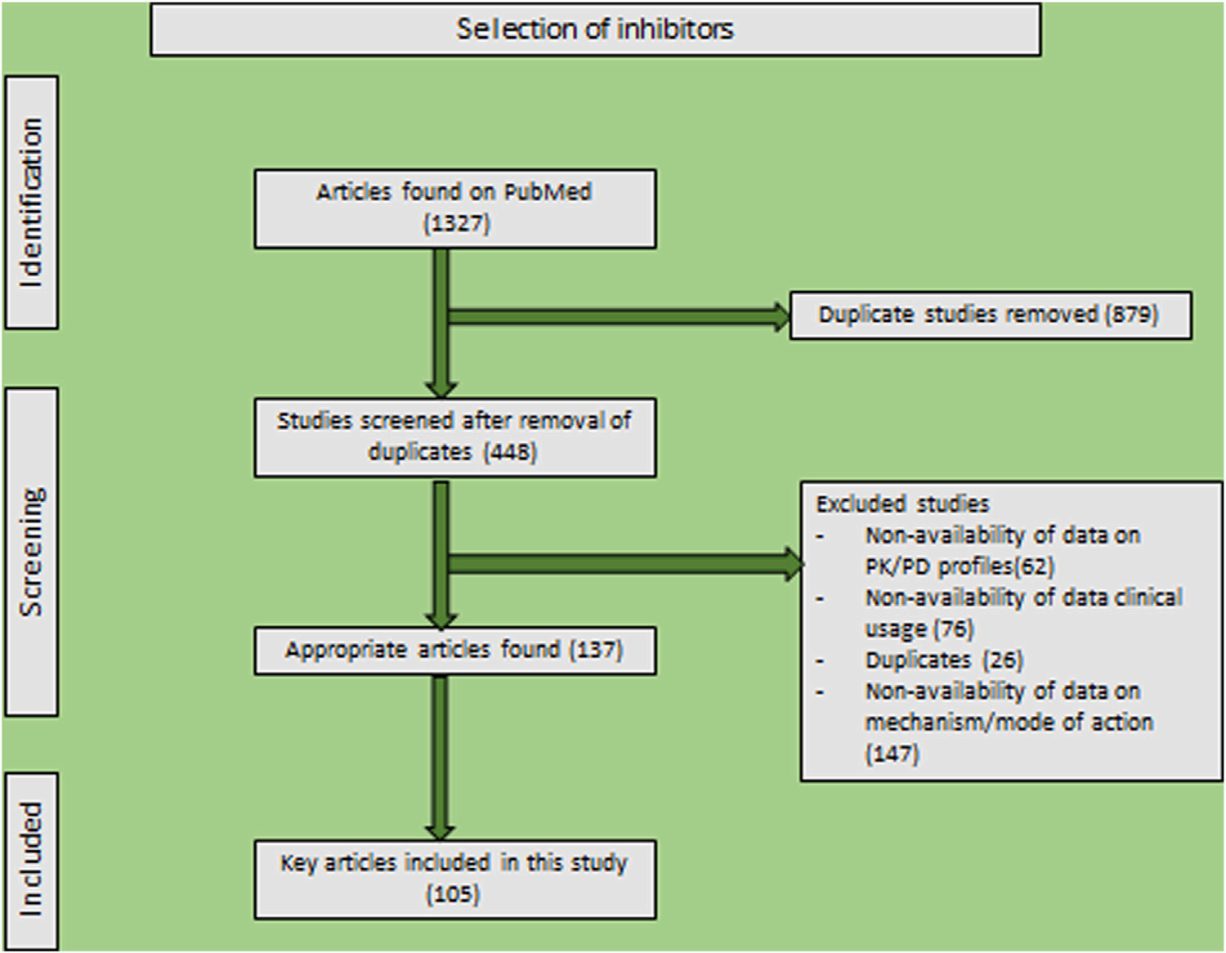
PRISMA statement for reporting systematic reviews and meta-analyses of research that assesses β-lactamase inhibitors.

This equation describes reversible inhibitors at equilibrium constant (Ki), equal to the *k*_-1_/*k*_1_ rate constant value, that would be independent of substrate concentration and reflect inhibitor affinity. Irreversible inhibitors are superior to reversible inhibitors due to their ability to prevent enzymes from functioning excessively (Bush, 1988; Copeland, 2005). Following this brief introduction, this review will delve into the mechanisms, structures, and therapeutic applications of a variety of β-lactam inhibitors. A comprehensive evaluation was made using the criteria presented here (Figure 1). A total of 1,327 articles were retrieved by an initial search. Following extensive pre-screening for duplicates, abstracts, and titles, 448 full-text articles were analyzed. Considering adequate information on inhibitors (including clinical studies, modes of action, and pharmacodynamic and pharmacokinetic profiles), 311 articles were excluded—125 were included in the final analysis.

## Avibactam

Avibactam is a β-lactamase inhibitor that was originally described in 2003 (Lagacé-Wiens et al., 2014). It inhibits class A and C β-lactamases, except for the synthesis of class C β-lactamases in *Enterobacter cloacae*. When administered independently, it exhibits minimal activity against a majority of organisms and has moderate activity on *E. coli*. A combination of avibactam and other β-lactam antibiotics restores antibacterial activity against class A and C β-lactamases, producing pathogens (Bennett et al., 2014).

Structure and mode of action: Chemically, avibactam is known as [(2S, 5R)-2-carbamoyl-7-oxo-1,6-diazabicyclo [3.2.1]octan-6-yl] hydrogen sulfate—molecular formula C_7_H_11_N_3_O_6_S. Its molecular mass is precisely 265.25 g/mol. Avibactam differs structurally from the rest of the utilized β-lactamase inhibitors in that it lacks a β-lactam ring (Ehmann et al., 2012). C7 carbonyl mimics the β-lactam carbonyl of cephalosporins, and sulfate at C6 is similar to the carbonyl group of ceftazidime at C4 (Shirley, 2018) (PubChemID: 9835049).

**FIGURE 2:**
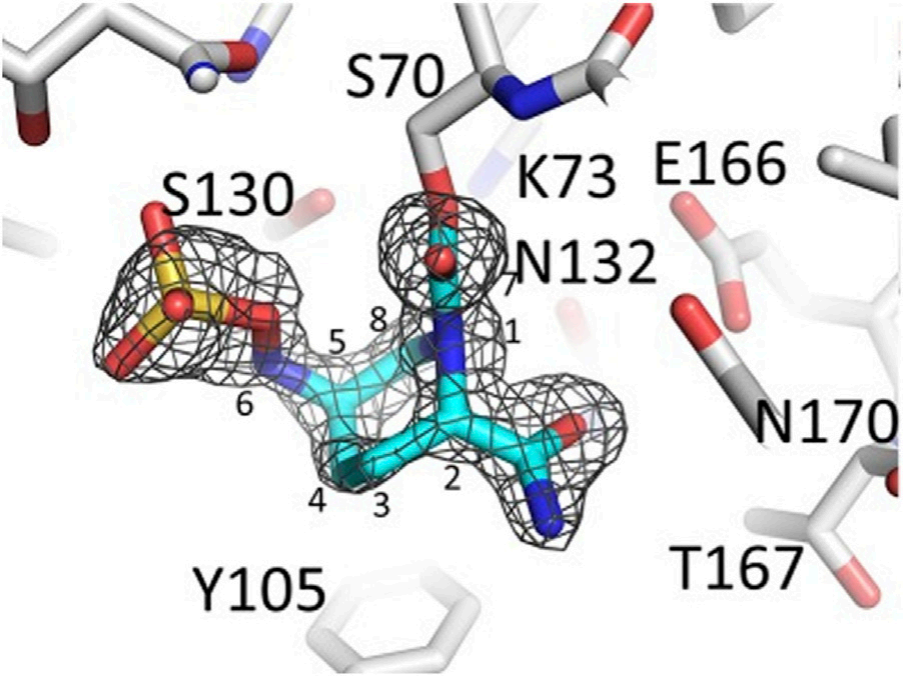
Representation of β-lactamase-avibactam complex. For the ligand outlined at 3.25σ, the difference in electron density between |Fo| and |Fc| is shown. In this blue stick figure representation, avibactam has been covalently bonded. (Adapted from Krishnan et al., 2015).

Avibactam has a unique mechanism; the process is reversible, unlike other β-lactamases. Though the strategy includes similar stages such as covalent binding (Figure 2) to the active site, the formation of an acyl-enzyme intermediate while unlocking the diaza-bicyclo octane ring structure thereby terminates β-lactam hydrolysis (Lahiri et al., 2013); hydrolysis is negligible in avibactam (Ehmann et al., 2012).

Pharmacodynamics: The pharmacodynamics (PD) of avibactam have been examined a number of times, most often in combination with ceftazidime. No growth was observed after treating an ampicillinase C (AmpC)-producing *E. cloacae* isolate and a *Klebsiella pneumoniae* Carbapenemase (KPC)-producing *K. pneumoniae* isolate with .1 g/mL of ceftazidime and avibactam (Levasseur et al., 2012). Apart from this, avibactam ≥2 μg/mL inhibited the growth of a plasmid-mediated cefotaximases (CTX-M-15)-producing *E. coli* and an AmpC-producing *E. cloacae*, while avibactam 2–4 μg/mL inhibited a KPC-producing *K. pneumoniae* (Lagacé-Wiens et al., 2014).

Clinical use: Infections caused by gram-negative bacteria, such as urinary tract infections, intra-abdominal infections, and hospital-acquired pneumonia, are treated with avibactam (Shirley, 2018). They are effective not only against gram-negative infections but also against a wide range of ESBL, AmpC, KPC, and OXA-48-producing Enterobacteriaceae and multidrug-resistant (MDR) *P. aeruginosa* isolates, except metallo-β-lactamase (MBL)-producing strains and *A. baumannii* spp. To treat a wide range of challenging diseases, avibactam-ceftazidime is utilized; however, a few resistant strains have also been discovered (Shirley, 2018; Papp-Wallace et al., 2020).

## Captopril

Captopril is a metallopeptidase enzyme that was developed in the 1970s and is used to treat hypertension by mirroring the actions of the angiotensin-converting enzyme (Akif et al., 2010). Scientists have found that captopril may bind to prosthetic groups and stop the enzyme from catalyzing (Zhao et al., 2021).

Structure and mode of action: Chemically, captopril is known as (2S)-1-[(2S)-2-methyl-3-sulfanylpropanoyl] pyrrolidine-2-carboxylic acid—molecular formula C_9_H_15_NO_3_S. Its molecular mass is precisely 217.29 g/mol. Crystalline powder is its most common form, with a melting temperature of 103°C–104°C and a pK_a1_ = 3.7, and a pK_a2_ = 9.8 (PubChem ID: 44093). It has a thiol ring, a free sulfhydryl group that serves as a zinc coordinating group, and two residues at P1’ and P2’ (Akif et al., 2010). Captopril is a metallo-β-lactamase inhibitor that inhibits through either metal or covalent binding (Yusof et al., 2016; Ju et al., 2018). Metal binding inhibition works by removing the metal ion from the enzyme or active site, or by forming a complex with protein residues that prevent antibiotics from binding (Brem et al., 2016).

Pharmacodynamics: Captopril was found to be effective *in vitro* against active-on-imipenem β-lactamase (IMP-1), Verona integron-borne metallo-β-lactamase (VIM-2), Sao Paulo metallo-β-lactamase (SPM-1), and New Delhi metallo-β-lactamase (NDM-1)-producing organisms such as *E. coli*, *K. pneumoniae*, *S. marascens*, and *P. aeruginosa*. When compared to L and D captopril, D captopril was found to be more effective, with side effects such as loss of taste and skin rash. Finding clinically-relevant MBL inhibitors is a formidable challenge; despite L-captopril being a well-studied ACE-2 inhibitor, there have been no reports of selectivity for human MBL-fold enzymes (Brem et al., 2016).

## Clavulanic acid

Clavulanic acid is a β-lactamase inhibitor isolated from *Streptomyces clavuligerus* and is most commonly used in association with β-lactamase drugs to treat β-lactamase resistance (Brown et al., 1979; Pruess and Kellett, 1983; López-Agudelo et al., 2021). *S. clavuligerus* is a gram-positive, sporing, and filamentous bacterium with a high ability to produce, as secondary metabolites, two classes of β-lactam compounds: those containing sulfur, and oxygen (Vanli, 2010). Isopenicillin N, desacetoxycephalosporin C, and cephamycin C are examples of sulfur-containing β-lactam compounds with antibiotic activity. Clavams are oxygen-containing β-lactam compounds that include clavulanic acid and other similar chemicals (Okamura et al., 1977; Kenig and Reading, 1979; Thai et al., 2001).

Structure and mode of action: Chemically, clavulanic acid is known as (2R,3Z, 5R)-3-(2-hydroxyethylidene)-7-oxo-4-oxa-1-azabicyclo [3.2.0]heptane-2-carboxylic acid—molecular formula C_8_H_9_NO_5_. Its molecular mass is precisely 199.16 g/mol. It is usually solid, with a boiling point of 545.8°C and a melting temperature of 117.5°C–118°C, as well as a pK_a_ of 2.7. Structurally, clavulanic acid contains a β-lactam ring lacking an acylamino side chain and an oxazolidine ring with O at C4 and 2-hydroxy ethylidene at the C2 position (PubChem ID: 5280980).

**FIGURE 3:**
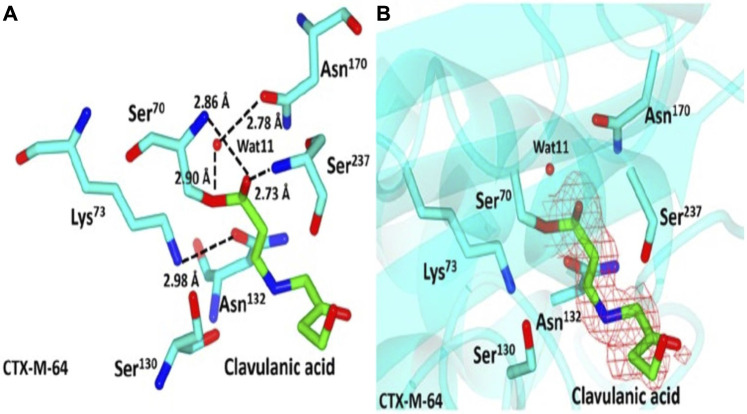
Representation of CTX-M-64-clavulanic acid complex. **(A)** Representation of CTX-M-64 clavulanic acid interactions, showing residue around the binding pocket as cyan sticks with atoms colored according to atom type. Clavulanic acid is shown here in green, with the various atoms indicated by their respective colors. **(B)** Red shows the contoured 2FoFc map over clavulanic acid in the CTX-M-64 pocket. Green sticks show clavulanic acid, while the cyan sticks show residues close to the binding domain. (Adapted from Cheng et al., 2019).

Clavulanic acid is a clavam metabolite that contains a β-lactam linked to an oxazolidine. Despite clavam metabolites having identical structures, their activity differs from that of clavulanic acid. Clavams with 3S, 5S stereochemistry have antibacterial activity, whereas clavulanic acid with 3R, 5R stereochemistry inhibits β-lactamases but has a low antimicrobial effect (Sydor and Challis, 2012). Clavulanic acid’s C7 carboxyl group forms a hydrogen bond with the active site S70 of β-lactamase (Figure 3), thereby favoring the formation of a stable acyl intermediate through a nucleophilic attack. Following the imine intermediate formed during the opening of the five-membered oxazolidine ring, the inhibitor linearizes and generates cis-enamine, which is further isomerized to the more stable trans-enamine *via* isomerization. This covalent acylation irreversibly inhibits the β-lactamase enzyme after several hours (Chen and Herzberg, 1992; Padayatti et al., 2005).

Pharmacodynamics: Minute doses of clavulanic acid are sufficient to inhibit the β-lactamase of common pathogens such as *H. influenzae* (.12 μg/mL) and *M. catarrhalis* (.01–.05 μg/mL) (Cooper et al., 1990). *In vitro* investigation of *S. pneumoniae* has revealed that clavulanic acid with β-lactams affects β-lactamase activity by interacting with penicillin-binding proteins (PBP), causing premature lysis and hypersensitivity to lysozyme, which results in major alterations to the cell wall (Severin et al., 1997).

Clinical use: Clavulanic acid is combined with amoxicillin in therapy (Uto and Gerriets, 2021). Drug trials have revealed that they are cost-effective in treating post-surgery infections, intra-abdominal infections, brain abscesses, diabetic foot infections, and pulmonary infections (Huttner et al., 2020). The combination is effective against gram-positive bacteria like *Staphylococcus epidermidis*, *Streptococcus pneumoniae*, *Enterococcus faecalis*, *Strep. pyogenes*, penicillin-methicillin-macrolide resistant strains, and gram-negative bacteria like *H. influenzae*, *Moraxella catarrhalis*, and *H. parainfluenzae* (Huttner et al., 2020; Uto and Gerriets, 2021).

## Durlobactam

Durlobactam, a diazabicyclooctane (DBO) β-lactamase inhibitor, has a wider range of action than existing inhibitors suited for intravenous administration (Shapiro et al., 2021). Durlobactam is an effective inhibitor that may be utilized for treating infections caused by *A. baumannii*. Durlobactam-sulbactam has potential as a supplement to existing pharmaceutical sources (Seifert et al., 2020).

Structure and mode of action: Chemically, durlobactam is known as [(2S, 5R)-2-carbamoyl-3-methyl-7-oxo-1,6-diazabicyclo [3.2.1]oct-3-en-6-yl] hydrogen sulfate—molecular formula C_8_H_11_N_3_O_6_S. Its molecular mass is precisely 277.26 g/mol. Durlobactam’s structure contains a ring of diazocyclooctene with a carbamoyl group, a methyl group, and sulfate, which is directly associated with the cyclo-octene ring (PubChem ID: 89851852).

**FIGURE 4:**
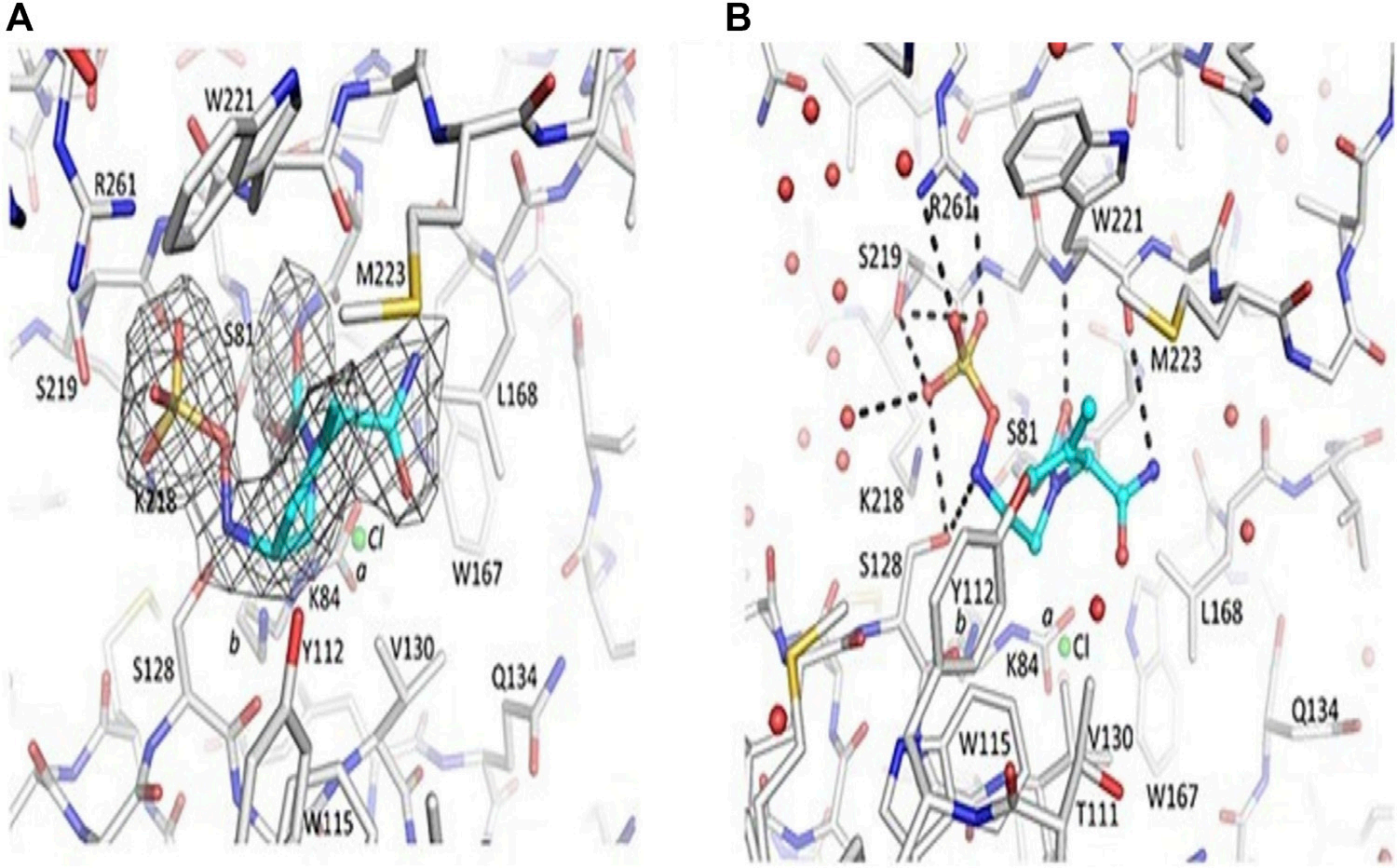
Representation of OXA-24/40-durlobactam complex. Durlobactam is also known as ETX2514. **(A)** ETX2514|Fo| |Fc| was eliminated from OXA-24/40 refinement and map calculations. Carbon atom sticks are used to symbolize the inhibitor (blue) and protein (gray). Three contours of electron density. The K84 side chain is carbamylated and non-carbamylated, with .6 and .4 occupancy conformations labeled as “a” and “b”, respectively. Additionally, the active site was optimized to contain a chloride ion with a .4 occupancy, represented by a green sphere labeled “Cl”. **(B)** ETX2514 and OXA-24/40 hydrogen bonding. Red spheres show water molecules, while green spheres show a chloride ion that is only partly filled. (Adapted from Barnes et al., 2019).

Durlobactam is known to have broader activity and is currently available on the market since it inhibits all classes of β-lactamase except class B. In terms of potency, durlobactam outperforms avibactam (Durand-Réville et al., 2017), with which it bears some resemblance: it attacks and modifies the enzyme in the serine active site, leading to the establishment of a covalent bond (Figure 4). As acylation proceeds, the ring is reformed, the inhibitor is released, and the sulfated amine can recyclize onto carbamate, which no longer reacts with β-lactamase (Shapiro et al., 2017). In gram-negative organisms, durlobactam gains access through outer membrane porins (OmpA), and OmpA deletion results in resistance to durlobactam (Isler et al., 2018).

Pharmacodynamics: When combined with sulbactam, durlobactam was effective in patients with complicated urinary tract infections. In addition, the pharmacokinetic-pharmacodynamic results using a dosage of 1,000 mg delivered every 6 h are being examined in phase 3 clinical trials (Sagan et al., 2020). Sulbactam–durlobactam is effective *in vivo* against extensively drug-resistant (XDR) A*. baumannii* clinical isolates in a variety of thigh and lung murine infection models, with MIC values ranging from .5/4 to 4/4 mg/L (Durand-Réville et al., 2017). Recent research in China has shown that durlobactam is effective against *A. baumannii* clinical isolates, with investigations ongoing (Yang et al., 2020).

Clinical use: Durlobactam, a new inhibitor used to treat *Acinetobacter* infections, is potent against serine β-lactamases of classes A, C, and D, as well as carbapenem-resistant strains (Shapiro et al., 2021), with phase 3 studies are still ongoing. The combination of sulbactam and durlobactam does not have any serious side effects. The side effects are mild to moderate, but healthy individuals have been able to handle them (Sagan et al., 2020). Sulbactam–durlobactam is also employed in the treatment of chronic infections (Wannigama et al., 2021).

## Enmetazobactam

Enmetazobactam, an N-methylated derivative of tazobactam, is known to inhibit members of all serine β-lactamase classes without major structural shattering (Lang et al., 2022). Enmetazobactam is a penicillin-derived sulfone specifically known to inhibit *Enterobacterales*-producing class C and D carbapenemases (Crandon and Nicolau, 2015; Morrissey et al., 2019).

Structure and mode of action: Chemically, enmetazobactam is known as (2S,3S, 5R)-3-methyl-3-[(3-methyltriazol-3-ium-1-yl)methyl]-4,4,7-trioxo-4lambda6-thia-1-azabicyclo [3.2.0]heptane-2-carboxylate—molecular formula C_11_H_14_N_4_O_5_S. Its molecular mass is precisely 314.32 g/mol. It contains a β-lactam ring with cyclic thiopentane derived from triazole (PubChem ID: 23653540). Enmetazobactam is a zwitter ion with a structure similar to tazobactam except for the methyl group at the triazole ring, which strengthens drug activity (Papp-Wallace et al., 2018a). The penam structure opens to react with the enzyme’s active site, forming a stable enzyme acyl complex that protects from hydrolysis. It inhibits serine β-lactamase and its production in bacterial species. Enmetazobactam is known to cause irreversible inhibition (Lang et al., 2022).

Pharmacodynamics: Enmetazobactam and cefepime’s effects on Enterobacteriaceae and *Pseudomonas aeruginosa* isolates were tested *in vitro*. It was found that enmetazobactam (8 μg/mL) lowered the MIC90 of cefepime, from 0.5 to 0.25 μg/mL for *Enterobacter* aerogenes, 16 to 1 μg/mL for *Enterobacter cloacae*, 16 to 0.12 μg/mL for *Escherichia coli*, and >64 to 0.5 μg/mL for *Klebsiella pneumoniae*, though it did not improve cefepime activity in *P. aeruginosa* isolates (Morrissey et al., 2019). While other studies into murine neutropenic thigh models reported that it restored cefepime effectiveness in all isolates, their pharmacodynamic target was greater than enmetazobactam MIC (Bernhard et al., 2020).

Clinical use: Enmetazobactam is used to treat urinary tract infections and nosocomial infections because it is effective against a limited number of enzymes from class A, C, and D β-lactamases (Johnson et al., 2020). They are effective against gram-negative bacteria. Furthermore, ongoing phase 3 studies have shown promising findings (Gallagher, 2020).

## ETX0282

ETX0282 is the prodrug of ETX1317, a DBO β-lactamase inhibitor (Durand-Reville et al., 2020) with an endocyclic carbon–carbon double bond and a fluoroacetate activation group that inhibits class A, C, and D serine β-lactamases (Miller et al., 2020).

Structure and mode of action: Chemically, ETX0282 is known as propan-2-yl (2R)-2-[[(2R, 5R)-2-carbamoyl-4-methyl-7-oxo-1,6-diazabicyclo [3.2.1]oct-3-en-6-yl]oxy]-2fluoroacetate—molecular formula C_13_H_18_FN_3_O_5_. Its molecular mass is precisely 315.30 g/mol. ETX0282 contains diazo cyclo-octane with fluoroacetic acid and carbamoyl group (PubChem ID: 146170992).

**FIGURE 5:**
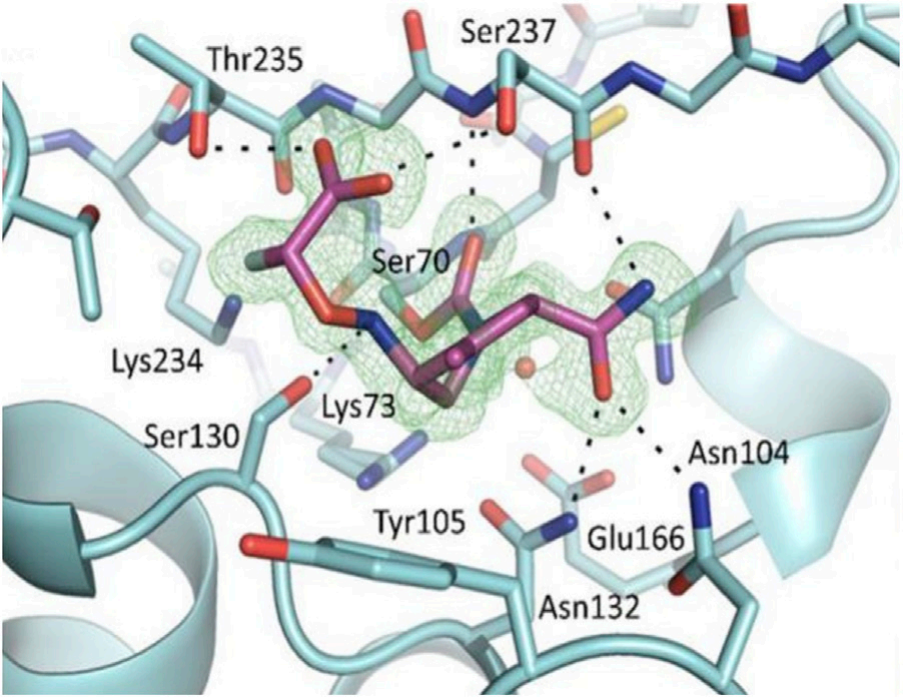
Representation of β-lactamase- ETX0282 complex. At 1.28 resolution, the complex crystal structure of ETX1317 with CTX-M-14 β-lactamase has been determined (PDB code 6VHS). Green contours on this impartial Fo–Fc map have a depth of 3 σ. In this illustration, the ligand is shown in purple, and the protein in blue. The ligand-protein hydrogen bond is shown as a black dashed line. A red sphere represents the catalytic water. (Adapted from Durand-Reville et al., 2020).

ETX0282’s structure and mechanism are analogous to those of other DBOs. It is a temporal inhibitor in which the urea ring S70 forms the covalent bond between the active site of the enzyme forming the acyl-enzyme complex; the oxyanion hole formed from the amide group of S70 and S237 is occupied by carbonyl oxygen (Figure 5). Due to the planarity of the C-C double bond, ETX1317 acquires a half-chair conformation upon ring opening, allowing the methyl group to migrate up and engage hydrophobically with Y105. This conformation causes the recyclization of the urea ring and β-lactamase recyclization (Lahiri et al., 2013).

Pharmacodynamics: In PK/PD studies, ETX1317 restored CPDP (cefpodoxime proxetil) activity to 1 μg/mL (CPDP), but the inhibitory action was not evident when ETX1317 was used alone, despite CPDP surpassing 50% T > MIC and ETX1317 passing a concentration twice the MIC for 60% of the dosing period. Allometric scaling of animal PK suggests that, at fixed ETX0282 and CPDP oral dosage, ETX1317’s clinical kinetics would be similar to that of cefpodoxime (O’Donnell et al., 2020).

Clinical use: ETX0282 and cefpodoxime are being developed as oral therapy for infections caused by MDR gram-negative organisms and carbapenem-resistant Enterobacteriaceae (CRE). Only a few people have experienced mild to moderate vomiting in the phase 1 trials since it was readily absorbed and had no drug–drug interactions (Mass, 2019). Phase 3 studies are now being conducted (NCT03491748).

## Nacubactam

Nacubactam, a potent β-lactamase inhibitor, inhibits penicillin-binding protein 2 in Enterobacteriaceae as well as classes A, C, and certain class D β-lactamases. Nacubactam suppresses β-lactamases to prevent co-administered drugs from hydrolyzing (Mallalieu et al., 2020). Nacubactam is effective against *Pseudomonas aeruginosa*, *Klebsiella pneumoniae*, and *Enterobacter cloacae* (Asempa et al., 2019; Hagihara et al., 2021).

Structure and mode of action: Chemically, nacubactam is known as [(2S, 5R)]-2-(2-aminoethoxycarbamoyl)-7-oxo-1,6-diazabicyclo [3.2.1]octan-6-yl]hydrogen sulfate—molecular formula C_9_H_16_N_4_O_7_S. Its molecular mass is precisely 324.31 g/mol. Its structure contains a diazo cyclooctane ring with an aminoethoxy carbomoyl group with a sulfated end (PubChem ID: 73386748).

**FIGURE 6:**
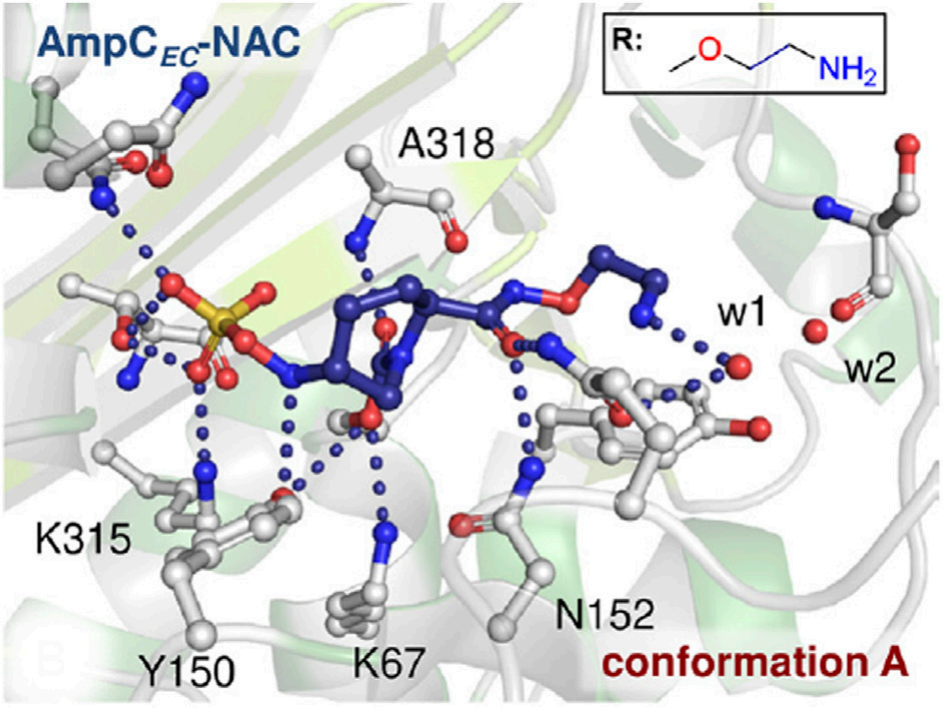
Representation of AmpC-nacubactam complex. Active regions of AmpC-nacubactam complex where nacubactam was modeled in a single conformation A in which N sulfate is close to Y150. (Adapted from Lang et al., 2021).

Nacubactam is a non-β-lactam inhibitor; it enhances the effectiveness of antibiotics when taken in combination, in addition to having antibacterial qualities (Hagihara et al., 2021). Nacubactam, like other β-lactam inhibitors, is known to shield β-lactams but more research is needed to determine exactly how they work (Livermore et al., 2015a). Following nacubactam binding, S64 is changed in a way that does not significantly affect anything. The reaction of nacubactam produced open ring products, with the piperidine ring assuming a chair conformation (Figure 6) and the carbamoyl-carbonyl oxygen positioned to engage with the backbone NH of A318. Through polar interactions with N346, T316, and K315, the N-sulfate group is 188 securely fixed in the active site (Lang et al., 2021).

Pharmacodynamics: In the pharmacodynamics study of Hagihara et al., 2021, strains that were treated with nacubactam and β-lactams such as aztreonam, cefepime, and meropenem showed antimicrobial activity on carbapenem-resistant Enterobacterales (CRE) *E. cloacae* and *K. pneumoniae*. On the other hand, meropenem in combination with nacubactam was ineffective against *K. pneumoniae* and CRE, resulting in low MIC (2 μg/mL); it was not effective against IMP-producing organisms (Livermore et al., 2015a; Livermore et al., 2015b; Morinaka, et al., 2015). Research on nacubactam combination therapy for CRE-caused pneumonia has shown promising results in a PD trial; however, only a few classes of carbapenemases have been examined (Yamamoto et al., 2017). Although CRE infections are becoming more common and harder to treat, this may open up new antimicrobial treatment options for CRE-caused pneumonia (Koizumi et al., 2018).

Clinical use: Nacubactam is used to treat infections like nephrotoxicity, β-lactam antibiotic-caused acute tubular necrosis, glomerulonephritis, and acute interstitial nephritis (Mallalieu et al., 2020). When combined with aztreonam, nacubactam shows a stronger antibacterial effect against MBL-producing Enterobacteriaceae with limited activity on *Pseudomonas* spp. and anaerobes (Mushtaq et al., 2019). Additional research indicates that the combination of meropenem and nacubactam is effective against carbapenem-resistant *K. pneumoniae* and MBLs (Carcione et al., 2021).

## Relebactam

Relebactam, a non-β-lactam inhibitor based on avibactam, was formerly known as diazabicyclooctane (DBO) (Stachyra et al., 2010). In the presence of imipenem-cilastatin, it is effective against both class A and C β-lactamases. The two together offer a high degree of safety. Relebactam, when combined with imipenem, restores imipenem’s effectiveness against a variety of imipenem-resistant bacteria, including Enterobacterales, that produce ESBL, AmpC, and KPC (Iyer 2022).

Structure and mode of action: Relebactam is chemically known as [ (2S, 5R) -7-oxo-2-(piperidin-4-ylcarbamoyl)-1,6-diazabicyclo [3.2.1]octan-6-yl]hydrogensulfate—chemical formula C_12_H_20_N_4_O_6_S. Its molecular mass is precisely 348.38 g/mol (PubChem ID: 44129647). It has a urea core structure similar to that of avibactam’s β-lactam ring and is highly reactive, which improves inhibition (Stachyra et al., 2010; Lahiri et al., 2013). The sole difference between relebactam and avibactam is the insertion of a piperidine substituent at position 2 of the carbamoyl group, which keeps cations and cell outflow intact (Mangion et al., 2011; Bhagunde et al., 2012).

**FIGURE 7:**
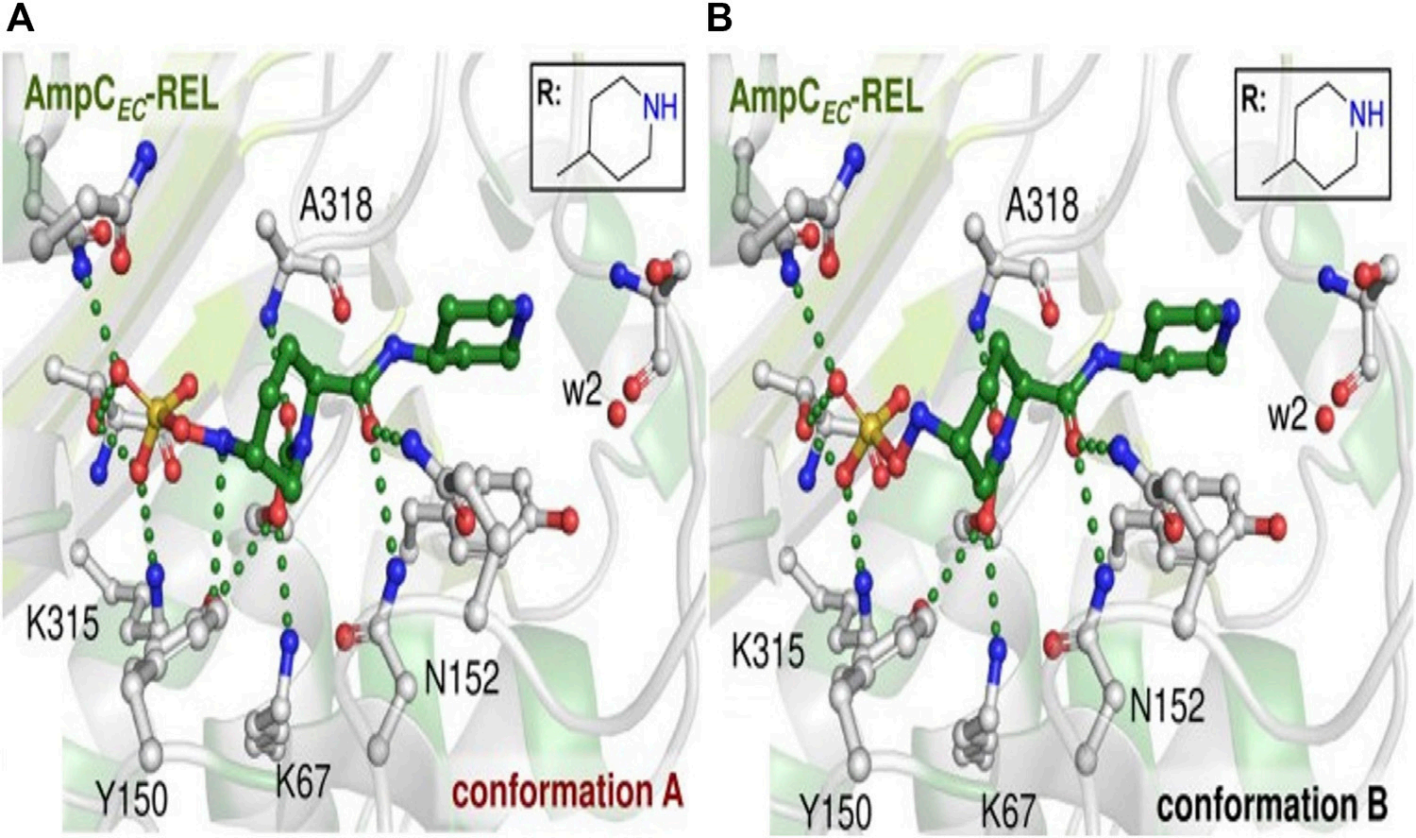
Representation of AmpC-relebactam complexes. Active region of AmpC-relebactam complexes where relebactam was modeled in N-sulfated conformations **(A,B)**. (Adapted from Lang et al., 2021).

Relebactam functions as a suicide inhibitor, resulting in irreversible inhibition (Lahiri et al., 2013). During acylation, a urea ring formed between C7 carbonyl and serine residues, resulting in intermediates that are stabilized by an aminoxy sulphate group through a hydrogen bond with the catalytic site (Figure 7). Relebactam further undergoes deacylation, resulting in active β-lactamase (Papp-Wallace et al., 2018b).

Pharmacodynamics: An *in vitro* study of the hollow fiber model, including *K. pneumoniae*, *E. coli*, *S. marcescens*, and *P. aeruginosa*, used imipenem and relebactam in a 2:1 ratio MIC, which was greater than the approved efficacy (>40%) (Mouton et al., 2000; Wu et al., 2018). Additional investigation demonstrated that MIC was linked with 2-log kill when a dose of imipenem–relebactam (500:250) was given every 6 h (Bhagunde et al., 2019).

Clinical use: Relebactam is used in the treatment of severe urinary tract and severe intra-abdominal infections (Campanella and Gallagher, 2020). According to the RESTORE-IMI 1 and -IMI 2 phase III clinical studies, relebactam is effective against pathogens such as atypical *Mycobacteria* spp. and non-imipenem-sensitive strains responsible for hospital-acquired bacterial pneumonia and ventilator-associated pneumonia. Relebactam is effective against carbapenem-resistant *P. aeruginosa*, ESBL, carbapenem-resistant Enterobacteriaceae (CRE), and gram-negative OXA producers (Karaiskos et al., 2022). Moreover, it is known to induce side effects such as nausea, diarrhea, and headaches (Campanella and Gallagher, 2020; McCarthy, 2020).

## Sulbactam

Sulbactam/ampicillin, a 1987 US-developed β-lactam/β-lactamase-inhibitor combination for intravenous and intramuscular use, irreversibly inhibits several bacterial β-lactamases but has limited antibacterial efficacy (Betrosian and Douzinas, 2009). The β-lactam ring in semi-synthetic penicillinate sulfone sulbactam is derived from 6-aminopenicllanic acid (Enna and Bylund, 2007). Researchers investigated how 14 bacterial and fungal species from all four Ambler classes broke down the drug sulbactam. Even within each class—A, B, C, and D—reaction kinetic constants differed (Shapiro et al., 2017). The drug is authorized as a first-line treatment for a wide variety of community-acquired illnesses in both children and adults. Due to the intrinsic activity of sulbactam, the combination may be regarded as especially effective against *Acinetobacter baumannii* infections (Betrosian and Douzinas, 2009).

Structure and mode of action: Chemically, sulbactam is known as (2S, 5R)-3,3-dimethyl-4,4,7-trioxo-4λ^6^-thia-1-azabicyclo [3.2.0]heptane-2-carboxylic acid—molecular formula C_8_H_11_NO_5_S Its molecular mass is precisely 233.24 g/mol. Sulbactam is usually solid, with a boiling point of 567.7°C ± 50.0°C, a melting temperature of 154°–157°C, and a pK_a_ of 2.62 ± .40. It is structurally similar to clavulanic acid, lacking an acylamino side chain, and has an oxazolidine ring with S at C4, a methyl group at C3, and a carboxylic group at the C2 position (PubChem ID: 130313).

**FIGURE 8:**
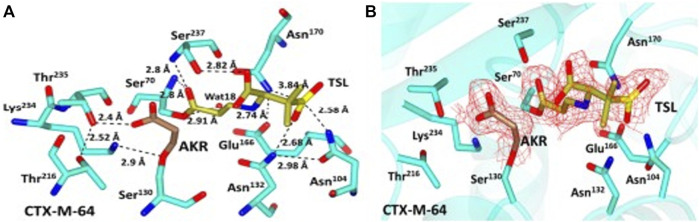
Representation of CTXM-64-sulbactam complex. **(A)** Sulbactam-CTX-M-64 pocket interactions. The sulbactam-CTX-M-64 crystal structure shows the residues in the region of the binding pocket as cyan sticks, with atoms colored according to their types. Gold represents acrylic acid (AKR) and trans-enamine intermediate (TSL), with each kind of atom colored differently. **(B)** Sulbactam derivatives are highlighted in red on the CTX-M-64 pocket’s 2FoFc map, which has been contoured at 3.0 σ around them. Cyan sticks represent residues around the binding pocket, whereas gold sticks represent AKR and TSL. (Adapted from Cheng et al., 2019).

Sulbactam is an irreversible inhibitor and semisynthetic (Carcione et al., 2021). Although individually it has minimal antimicrobial activity, it shows a synergistic impact on β-lactamase producers when combined with β-lactams (Enna and Bylund, 2007). Sulbactam’s β-lactam ring assists in the inactivation of β-lactamase by irreversibly binding. The actual mechanism is unknown but it is widely assumed that sulbactam is initially recognized as the normal substrate by β-lactamases and generates an acyl intermediate complex by interacting with the active site serine hydroxyl group (Figure 8). This complex undergoes further deacylation, tautomerism, and transamination with S130 so that the enzyme is irreversibly blocked (Rafailidis et al., 2007).

Pharmacodynamics: For sensitive strains and *E. coli*, 3 g of ampicillin-sulbactam every 6 h is sufficient. Lower doses are ineffective against the TIM2 strain but are effective against ATCC 25922 and EC1. Few *E. coli* strains cause a higher rate of durability in genitourinary tract infections, which may be due to low dosages of ampicillin-sulbactam or resistance mechanisms other than the β-lactamase production. It is thus unclear if ampicillin-sulbactam resistance leads to long-term clinical failure (Enna and Bylund, 2007).

Clinical use: Sulbactam is typically used in combination with ampicillin to treat skin and soft tissue illnesses, lower respiratory tract infections, intra-abdominal infections, diabetic foot infections, and in pediatrics. However, it is ineffective against *P. aeruginosa* (Lamp and Vickers, 1998). Sultamicillin has been shown in clinical trials to be clinically efficacious in adults and children against a variety of commonly encountered illnesses. Sulbactam is usually used in a 1:2 ratio with cefoperazone, demonstrating exceptional activity against *Enterobacteriaceae* spp., *P. aeruginosa*, and *A. baumannii*. However, a few resistant strains of *A. baumannii* have been discovered (Yang et al., 2018; Ku and Yu, 2021).

## Tazobactam

Tazobactam is a β-lactamase inhibitor that prevents the degradation of piperacillin by β-lactamases. When coupled with piperacillin, tazobactam expands the range of antibacterial activity against *Staphylococcus* spp., *Enterobacteriaceae* spp., *Haemophilus influenzae*, and *Bacteroides* species (Perry and Markham, 1999).

Structure and mode of action: Chemically, tazobactam is known as (2S,3S, 5R)-3-methyl-4,4,7-trioxo-3-(triazol-1-ylmethyl)-4λ^6^-thia-1-azabicyclo [3.2.0]heptane-2-carboxylic acid—molecular formula C_10_H_12_N_4_O_5_S. Its molecular mass is precisely 300.29 g/mol. Tazobactam is usually solid, with a boiling point of 77°C and a melting temperature of 140°C–147°C, as well as a pK_a_ of 2.1 (PubChem ID: 123630).

**FIGURE 9:**
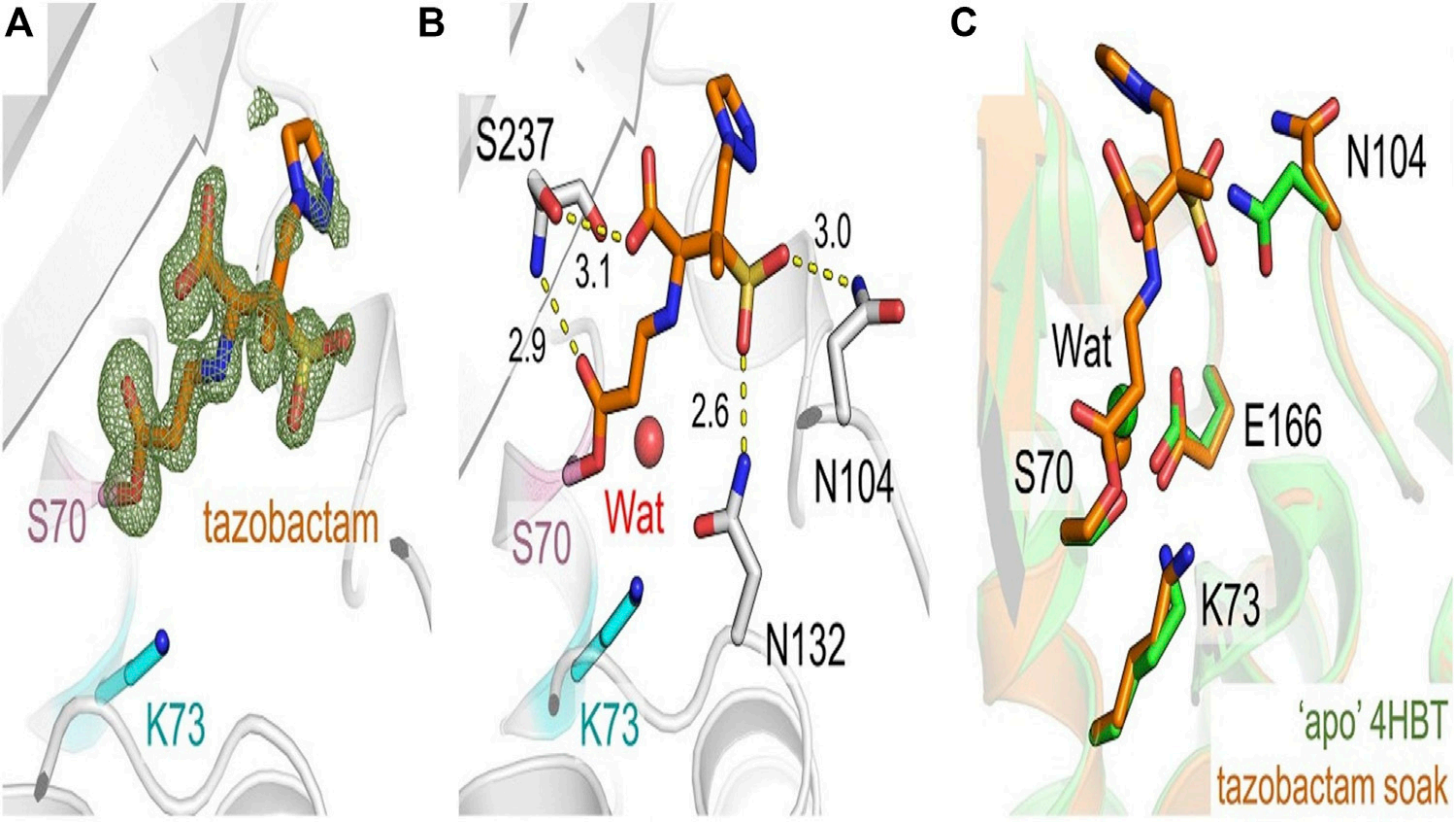
Representation of CTX-M-15-Tazobactam complex. Tazobactam is represented in orange sticks. Ser70 and Lys73 are represented by pink and gray sticks, whereas CTX-M-15 backbone is displayed in a neutral gray. Stick representation of the antibiotic tazobactam in orange. Fo-Fc electron density (green, 3) was determined **(A)** after tazobactam was eliminated from the equation. Distances in angstroms are identified in **(B)**, and interactions between tazobactam and residues in the CTX-M-15 active site are shown as yellow dashes. **(C)** Superposition of CTX-M-15: tazobactam onto the active site of unliganded apo CTX-M-15 (green, PDB code 4HBT [34]). Catalytic waters at the active site are shown as spheres with different colors for each protein. (Adapted from Hinchliffe et al., 2022).

Tazobactam belongs to penicillanic acid and is one of the exocyclic methyl hydrogens substituted by a 1,2,3-triazol-1-yl group (Tooke et al., 2019). It is derived from 6-aminopenicllanic acid. Tazobactam, an irreversible inhibitor, forms an imine acyl complex (Figure 9) by attacking the active site of the enzyme. This complex can go through any of the following processes: deacylation, liberating active enzyme and hydrolyzed product, irreversible damage by tautomerization, or degradation *via* sets of reactions (Yang et al., 1999). However, the inhibition is determined by the rate of deacylation and tautomerization (Drawz and Bonomo, 2010).

Pharmacodynamics: Tazobactam functions well with cefepime, ceftolozane, and piperacillin (Papp-Wallace et al., 2019). In cefepime-tazobactam, the threshold of tazobactam dosage is linked to net bacterial stasis and a drop in 1-log_10_ colony-forming unit (CFU) from 52.8% to 21.9%; the *in vitro* infection model requires more study (VanScoy et al., 2017). Piperacillin and tazobactam were also used to test patients, but the results did not meet pharmacodynamic goals (Bauer et al., 2012; Thabit et al., 2016).

Clinical use: Tazobactam is used in the treatment of appendicitis, skin infections, community-acquired nosocomial pneumonia, and gynecological infections. It can be used against gram-positive as well as gram-negative organisms. Nonetheless, tazobactam has adverse effects in individuals with hypersensitive reactions, renal impairment, bleeding manifestations, or individuals undergoing cytotoxic therapies. Common effects during tazobactam administration are gastrointestinal inflammation or fever (Perry and Markham, 1999; Sarkar et al., 2017).

## Taniborbactam

Taniborbactam is the first boronate inhibitor of all β lactamases classes (Hamrick et al., 2020; Liu et al., 2020). It is an extremely strong, gram-negative outer-membrane-penetrating inhibitor (Liu et al., 2020).

Structure and mode of action: Chemically, taniborbactam is known as (3R)-3-[[2-[4-(2-aminoethylamino) cyclohexyl] acetyl] amino]-2-hydroxy-3,4-dihydro-1,2-benzoxaborinine-8-carboxylic acid—molecular formula C_19_H_28_BN_3_O_5_. Its molecular mass is precisely 389.3 g/mol. Taniborbactam contains a basic benzoxaborinie ring with carboxylic acid as one side chain; the other side chain is the cyclohexane chain linked *via* the carbamoyl moiety. The cyclohexane has a side chain of amino ethanol amine (PubChem ID: 76902493).

**FIGURE 10:**
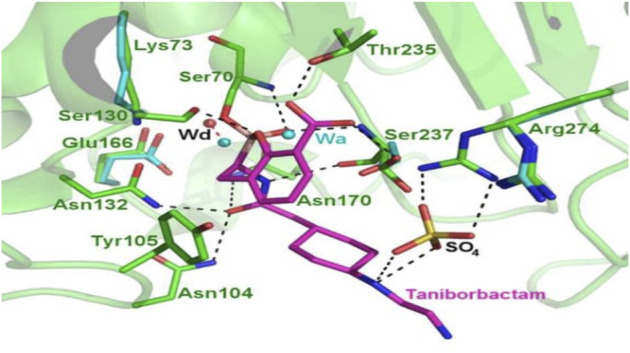
Representation of CTX-M-15-taniborbactam complex. CTX-M-15-taniborbactam binding to the active site, revealing the primary sites of contact between enzyme and inhibitor (magenta). Taniborbactam binds with many conserved residues, and the inhibitor displaces the deacylation water molecule (Wd) by 1.4Å. Both Wa and SO4 are components of crystallization buffer solution; Wa is acylation water. (Adapted from Hamrick et al., 2020).

Taniborbactam is a reversible inhibitor that covalently binds to the active serine site (S70) (Figure 10); due to this, the boron atom confers a tetrahedral conformation, imitating an intermediate, and interacts with N104, S130, N132, N170, and T235-conserved sites of β-lactamases—thus narrowing the active site inhibiting β-lactamase (Hamrick et al., 2020).

Pharmacodynamics: *In vitro* investigation has revealed that a combination of taniborbactam and cefepime (2 g/0.5 g q8h as a 2 h infusion) has significant effects on isolates where a 1-log reduction was described in substantially all isolates, with only a few showing a 2-log reduction and few reporting a 3-log10 reduction (Abdelraouf et al., 2020). Taniborbactam also recovers cefepime activity; the MIC of cefepime alone was 256 and 32 g/mL after taniborbactam was introduced; the cefepime MIC was 4 g/mL in both cases (Hamrick et al., 2020).

Clinical use: Taniborbactam, in combination with cefepime, is used to treat complex urinary tract infections such as acute pyelonephritis and hospital-acquired or ventilator-associated bacterial pneumonia. However, moderate side effects, including headache, nausea, dizziness, and diarrhea, have been reported (Dowell et al., 2021).

## Vaborbactam

The new β-lactamase inhibitor vaborbactam is based on a cyclic boronic acid pharmacophore with significant inhibitory efficacy against class A and C β-lactamases. To re-establish its effectiveness against *Klebsiella pneumoniae* carbapenemases, it has been co-formulated with meropenem (Jorgensen and Rybak, 2018). Combining with meropenem is thought to be a good way to treat severe infections caused by gram-negative bacteria (Lomovskaya et al., 2017). Vaborbactam’s boronic acid pharmacophore yields a boronic ester ring that imposes a favorable form for binding structure, resulting in improved efficacy (Bhowmick and Weinstein, 2020).

Structure and mode of action: Chemically, vaborbactam is known as 2-[(3R, 6S)-2-hydroxy-3-[(2-thiophen-2-ylacetyl)amino]oxaborinan-6-yl]acetic acid—molecular formula C_12_H_16_BNO_5_S. Its molecular mass is precisely 297.14 g/mol. It contains a thiophene ring derivatized with oxaborinane through a peptide bond between acetyl and amino groups, which is linked to an acetate group (PubChem ID: 77846445).

**FIGURE 11:**
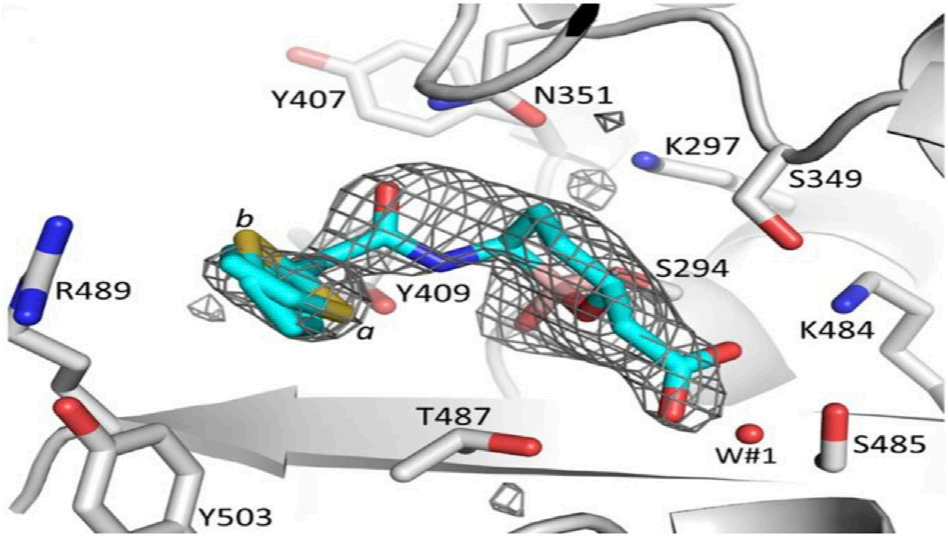
Representation of β-lactamase-vaborbactam complex. Vaborbactam is represented by spherical cyan carbon atoms. Vaborbactam’s covalent interaction to the catalytic S294 shown as a stick model with differential density contouring at the 2.75 level. (Adapted from Kumar et al., 2021).

Vaborbactam inhibits class A and C serine β-lactamases—specifically KPC β-lactamases. The presence of a 2-thienyl acetyl group in vaborbactam’s structure helps enhance the inhibitor’s effectivity (Hecker et al., 2015). Vaborbactam enters the organism *via* porins OmpK35 and OmpK36 on the outer membrane (Lomovskaya et al., 2017) and acylates the enzyme’s catalytic serine residue, leading to the formation of a complex (Figure 11). Vaborbactam initially forms a non-covalent complex, followed by a covalent interaction between the catalytic Ser residue of the enzyme and the boron atom of vaborbactam to form the enzyme inhibitor (EI*) complex. This reaction can be reversed because a water molecule can quickly break down the covalent bond between the catalytic serine residue and the boron atom to release vaborbactam (Tsivkovski et al., 2020).

Pharmacodynamics: There is a scarcity of published data on vaborbactam’s pharmacodynamics. It is usually utilized in meropenem combinations; MICs ranging from 0.06/8 to 64/8 mg/L were seen in isolates of Enterobacteriaceae that produce KPC β-lactamases. However, when experiments were conducted on a hollow-fiber model in a 2:2 ratio for every 8 h by 3-h infusion, a 6-log kill was achieved and resistance was silenced (Petty et al., 2018). Furthermore, the same dosage was investigated in a hollow-fiber model and a neutropenic murine thigh model; the hollow fiber exhibited the restoration of meropenem antimicrobial activity, whereas the murine thigh model was attributed to bacteriostasis and 1-log10 kill (Griffith et al., 2018).

Clinical use: Meropenem-vaborbactam’s pharmacokinetic properties were compatible and clinically tolerated in phase I studies (Wenzler et al., 2015; Griffith et al., 2016). Based on phase I studies, antibiotic-nonsusceptible gram-negative organisms (TANGO I and II) were targeted. In TANGO I, meropenem-vaborbactam surpassed piperacillin-tazobactam in patients with complicated urinary tract infections, including acute pyelonephritis, while TANGO II showed fewer side effects in patients with CRE infections (Petty et al., 2018).

## WCK-4234

WCK-4234 belongs to the diazabicyclooctanes inhibitor class, which is highly effective against class A and D β-lactamases (Mushtaq et al., 2017). Meropenem and WCK 4234 effectively function together. The latter increases carbapenem activity but does not inhibit metallo-β-lactamases (Iregui et al., 2019).

Structure and mode of action: Chemically, WCK4234 is known as sodium; [(2S, 5R)-2-(5-methyl-1,3,4-oxadiazol-2-yl)-7-oxo-1,6-diazabicyclo [3.2.1]octan-6-yl]sulfate—molecular formula C_9_H_11_N_4_NaO_6_S. Its molecular mass is precisely 326.26 g/mol. Its structure contains a diazo-octane ring with methyl, and aminosulfate groups (PubChem ID: 140620411).

WCK-4234 has been identified as a reversible inhibitor. WCK-4234 binds covalently to the active serine site (S70), leading to the formation of an acyl enzyme intermediate and inactivation of the enzyme; however, it halts degradation and restores itself. Compared to avibactam, WCK-4234 has a different desulfation process, but its effectiveness needs more study (Papp-Wallace et al., 2018a).

Pharmacodynamics: Although WCK-4234 increases drug function, it has insufficient antibacterial qualities (Mushtaq et al., 2017). It is effective against carbapenemases of classes A, D, and C, as well as class C enzymes (Iregui et al., 2019). The MIC50/90 was reported from *in vitro* studies against carbapenem-resistant *A. baumannii* isolates (no resistance genes detected) treated with meropenem and WCK-4234 (2 and 8 μg/mL) (Sader et al., 2017). However, they were less effective against OXA-23 (2/4 g/mL) and OXA-40 (4/8 g/mL) with MIC50/90 recorded (Mushtaq et al., 2017).

Clinical use: In preclinical trials, meropenem-WCK-4234 treatment for a neutropenic lung infection with multidrug-resistant OXA-23-producing A*. baumannii* has been observed to reduce bacterial load by 2.5 logs; however, WCK-4234 is yet to be tested in clinical studies (Papp-Wallace et al., 2018b).

## Xeruborbactam (QPX7728)

QPX7728 is a cyclic boronate-based β-lactamase inhibitor; it is effective against Enterobacterales class A and D carbapenemases as well as a variety of *Acinetobacter* carbapenemases (Lomovskaya et al., 2021) and class B β-lactamases (Tsivkovski et al., 2020). It is most effective as an inhibitor of organisms that produce carbapenemases (Nelson et al., 2020).

Structure and mode of action: Chemically, xeruborbactam is known as (1aR,7bS)-5-fluoro-2-hydroxy-1a, 7b-dihydro-1H-cyclopropa [c][1,2] benzoxaborinine-4-carboxylic acid—molecular formula C_10_H_8_BFO_4_. Its molecular mass is precisely 221.98 g/mol. Xeruborbactam contains benzoxaborinie, which has a carboxylic acid side chain with a twinning ring of fluorocyclohexane (PubChem ID: 140830474).

QPX7728 inhibitors are reversible; the boron atom in the inhibitor binds covalently to the active serine site (S70), forming a complex that inactivates the enzyme. However, the complex is dissociated and the enzyme is restored after a certain time (Tsivkovski et al., 2020). The critical interactions that contribute to the inhibitor’s high-affinity binding to MBLs are: carboxylic acid’s oxygen atom, boronate ester oxygen, and hydroxyl group ligand; direct 2 zinc ions at the active site; cap-generating lipophilic linkages with loop L65:V73 of the side chain; aid in the formation of the salt bridge (Lomovskaya et al., 2021).

Pharmacodynamics: QPX7728 in combination with meropenem shows promising *in vitro* study results against carbapenem-resistant *Acinetobacter* spp. (CRAB) with diverse resistance mechanisms, reducing the MIC_90_ of meropenem from >64 to 8 μg/mL and 4 μg/mL (Nelson et al., 2020). However, QPX7728 can also be used with ceftazidime, avibactam, ceftolozane-tazobactam, meropenem, vaborbactam, imipenem, and relebactam—all of which have been approved for clinical use against organisms like *Acinetobacter baumannii*, *Pseudomonas aeruginosa*, and *Enterobacterales* (Lomovskaya et al., 2021).

Clinical use: QPX7728 phase 1 trials are underway: it is known to inhibit KPC, NDM, VIM, and OXA β-lactamase-producing organisms such as *Klebsiella pneumoniae*, *P. aeruginosa*, *Enterobacteriaceae* spp., and *A. baumannii* (NCT04380207).

## Zidebactam

Zidebactam is known to inhibit PBPs and β-lactamases, and to synergize with them. It is a bicyclo-acyl hydrazide that inhibits class A, C, and D β-lactamases. Zidebactam, produced from a DBO architecture, was intended to increase PBP2 binding rather than antagonistic activity—to promote β-lactam activity. However, it also possesses considerable β-lactamase inhibitory action (Vázquez-Ucha et al., 2020).

Structure and mode of action: Chemically, zidebactam is known as [(2S, 5R)-7-oxo-2-[[[(3R)-piperidine-3-carbonyl]amino]carbamoyl]-1,6-diazabicyclo [3.2.1]octan-6-yl]hydrogen sulfate—molecular formula C_13_H_21_N_5_O_7_S. Its molecular mass is precisely 391.40 g/mol. Zidebactam contains diazocyclo-octane with a six-membered piperidine ring side chain that is attached *via* a carbamoyl group. The other branch is the sulfated side chain (PubChem ID: 77846445).

Zidebactam plays a vital role in gram-negative organisms by selectively binding to PBP2 with high affinity and inhibiting β-lactamase activity (Sader et al., 2017). It is covalently attached to S294 (Figure 12), and active residues of PBP interact with diacylhydrazide. This is achieved by reorganization. This diacylhydrazide moiety can act either as a hydrogen donor or an electron acceptor. These interactions between diacylhydrazides and the R1-group piperidine ring inhibit the β-lactamase and have an antibacterial property (Rajavel et al., 2021).

Pharmacodynamics: Zidebactam is now being studied in combination with cefepime at a dose of 2 g/1 g, infusing it every hour for 8 h; results indicate that it serves as both a β-lactamase inhibitor and a cefepime enhancer (Monogue et al., 2019). In an *in vivo* study in a murine lung infection model, the minimum elongation concentration (MEC) and minimum spheroplastation concentration (MSC) were determined and further split into several minimum inhibitory concentrations (MIC’s). These improved the *R* ^2^ values compared to the PD analysis MICs of cefepime and zidebactam, in addition to the PK/PD index being fT > 0.015x in *A. baumannii* isolates (Bhagwat et al., 2019). One of the most essential aspects of zidebactam’s pharmacodynamics is its enhancer property. The cefepime fT > MIC required to generate a 1-log10 colony-forming unit (CFU) kill against multidrug-resistant *A. baumannii* was dropped from 38.9% to 15.5% with zidebactam (Bhagwat et al., 2019).

Clinical use: Most *E. coli*, *K. pneumoniae*, *Citrobacter* spp., *Enterobacter* spp., *Serratia* spp., multidrug-resistant *P. aeruginosa*, *S. maltophilia*, and *Burkholderia* spp. that cause infections are treatable with zidebactam (Carcione et al., 2021). Meanwhile, a phase 3 trial for treating complicated urinary tract infections and acute pyelonephritis is currently active (NCT04979806).

## Complications

As explained above, there are both established and emerging inhibitors (Table 1). Creating a novel brand of inhibitor is a difficult task. Inhibitors with border spectrum action may be useful in addressing broad-spectrum resistance, like, for example, taniborbactam inhibits KPC, OXA, and most of the metallo-β-lactamases but not IMP β-lactamases. Another challenge is that certain inhibitors, such as WCK-4234, only have a mild bactericidal effect. The minute size of the active sites of metallo-β-lactamases result in a significant problem for the development of inhibitors for class B β-lactamases. Hydrogen bonding between the inhibitor and the target may help increase its selectivity, which is a problem in and of itself. Although the nature of polarity plays a significant role at the membrane-water interface, it is reasonable to conclude that the non-polar form of the inhibitors predominate within the membrane (owing to its hydrophobic nature)—neutral forms are thus preferable over charged ones (Zhu et al., 2017). Finally, a major hurdle in developing an efficient inhibitor is the emergence of microbial resistance to current pharmaceutical classes. There is also a need for greater study into the development of inhibitors for multidrug-resistant pathogens that may counteract the key mechanism whereby antibiotics avoid being digested by β-lactamases.

**TABLE 1:** Data on inhibitor classes, inhibitory mechanisms, and current clinical trial status.

	Inhibitors	Type of inhibitor	Type of inhibition	Phase trial status	Applicability	Reference
1	Avibactam	DBO	Reversible	Phase III^a^	GNO	Lahiri et al. (2013)
2	Clavulanic acid	β-Lactam	Irreversible	Phase III^a^	BGNP	Sydor and Challis (2012)
3	Captopril	Metallo-β-lactam inhibitor	Reversible	Phase II	BGNP	Zhao et al. (2021)
4	Durlobactam	DBO	Reversible	Phase III	GNO	Shapiro et al. (2021)
5	Enmetazobactam	Cyclic boronate inhibitor	Irreversible	Phase III	GNO	Lang et al. (2022)
6	ETX0282	DBO	Reversible	Phase I	GNO	Lahiri et al. (2013)
7	Nacubactam	Non-β-lactam	Reversible	Phase I	GNO	Lang et al. (2021)
8	Relebactam	Non-β-lactam	Irreversible	Phase III^a^	GNO	Stachyra et al. (2010)
9	Sulbactam	β-Lactam	Irreversible	Phase IV^a^	BGNP	Carcione et al. (2021)
10	Tazobactam	β-Lactam	Irreversible	Phase IV^a^	BGNP	Tooke et al. (2019)
11	Taniborbactam	Cyclic boronate inhibitor	Reversible	Phase I	GNO	Lui et al. (2020)
12	Vaborbactam	Cyclic boronate inhibitor	Reversible	Phase I^a^	GNO	Tsivkovski et al. (2020)
13	WCK-4234	DBO	Reversible	Preclinical trials	GNO	Papp-Wallace et al. (2018a)
14	Xeruborbactam	Cyclic boronate inhibitor	Reversible	Phase I	BGNP	Lomovskaya et al. (2021)
15	Zidebactam	DBO	Reversible	Phase III	BGNP	Rajavel et al. (2021)

^a^ Currently used clinically.

GNO, gram-negative organisms; BGNP, both gram-negative and positive organisms.

## Conclusion

One of the most pressing issues in modern public health is the spread of antibiotic-resistant bacteria. Antimicrobial resistance is a growing problem that raises serious concerns about the efficacy of β-lactam drugs. β-lactamase inhibitors can be used to combat β-lactamase-mediated antibiotic resistance. An inhibitor’s utility is enhanced when it can block activity without being reversed. Reversible inhibitors may reactivate the enzyme by breaking non-covalent interactions, whereas irreversible inhibitors which bind covalently are thus unaffected by the presence of alternative substrates. The metallo-β-lactamases are the most challenging to treat as their active site is located in shallow grooves. Taniborbactam, a metallo-β-lactamase inhibitor, lacks broad-spectrum activity, whereas captopril, another metallo-β-lactamase inhibitor, has an undesirable side effect. When it comes to inhibiting β-lactamases, clavulanic acid is the most effective inhibitor discovered thus far because it inhibits both gram-positive and gram-negative organisms with few side effects. Researchers should take into account the probability that their discoveries regarding β-lactamase inhibitors should have broader spectrum activity. In combating antimicrobial resistance, it will be useful to develop or modify inhibitors with irreversible action and reduce adverse effects by considering the polarity and pK_a_ of compounds. Resistance-developing bacteria to inhibitors have been found recently. Investigating the expression and alteration of trans-membrane proteins, which may be distributed *via* plasmids, may increase knowledge about the dissemination and evolution of inhibitor-resistant strains.

## Abbreviations

ESBL, extended-spectrum β-lactamase enzymes; AmpC, ampicillinase C; KPC, *Klebsiella pneumoniae* carbapenemase; MDR, multidrug-resistant; MBL, metallo-β-lactamase; PBP, penicillin-binding protein; IMP, active-on-imipenem; VIM, Verona integron-borne metallo-β-lactamase; SPM, Sao Paulo metallo-β-lactamase; NDM, New Delhi metallo-β-lactamase; OXA, oxacillinase; XDR, extensively drug-resistant; CRE, carbapenem-resistant Enterobacteriaceae; PD, pharmacodynamics; PK, pharmacokinetics; DBO, diazabicyclooctane; MEC, minimum elongation concentration; MSC, minimum spheroplastation concentration; MIC, minimum inhibitory concentration; CFU, colony forming unit; GNO, gram-negative organisms; BGNP, both gram-negative and positive organisms.
